# Around the Hospitals

**Published:** 1893-08-05

**Authors:** 


### AROUND THE HOSPITALS.

Notice to the Managers of Institutions.—Special space is now reserved for the insertion of notices of hospital meetings and festivals. The Editor requests that all notices may reach The Hospital Office, 428, Strand, at least one week before the date of each meeting.

**The Hospital for Sick Children,** Great Ormond Street.—The principal event of the year at the Children's Hospital has been the opening of the new wing in June by their Royal Highnesses the Prince and Princess of Wales. Such full accounts of this interesting ceremony appeared in all the papers and in our own columns that it is unnecessary to enlarge upon it now. The wing was furnished and occupied some time previous to the opening ceremony. The building fund is in urgent need of help. The original estimate had to be exceeded by £3 000, and the adverse balance stands at nearly £12,000. Help is not only needed in this direction. The general funds of the hospital require a substantial increase. Donations and subscriptions have been falling off, and legacies that have fallen in lately have been applied as income instead of increasing the endowment fund, as is desirable. The Great Ormond Street Hospital was, perhaps, at one time the most popular of all hospitals in the eyes of the public. Sympathy with sick children is a universal quality, and, therefore, there should be no difficulty in keeping up an unflagging interest in the institution. The last annual dinner, under Lord Halsbury's presidency, procured a substantial sum for the hospital. The new cots are the St. Petersburg cot, perpetually endowed by friends in memory of the late Miss Feild; and the *Pall Mall Budget* cot, supported by the child readers and others of that journal. During the year 1,281 in-patients and 21,095 out-patients were treated in the hospital. There have been a good many changes in the medical staff during the past year, including the resignation of the well-known children's doctor, Dr. Cheadle, after 25 years of excellent work. Dr. Cheadle remains on the consultants' staff.

**The Royal Portsmouth Hospital.**—The annual report of this hospital contains a paragraph which embodies a statement on which the management should be congratulated, it reads: "There is scarcely an item of income which does not show an increase, or of expenditure upon which there is not a decrease." First and foremost stands the increase of £124 on annual subscriptions, a most substantial and satisfactory addition to the funds of the hospital. Legacies have produced £1,236, and entertainments organised by the ex-Mayoress, Mrs. Scott-Foster, have materially aided the institution. The number of in-patients treated give an increase of 69, and out-patien's of 138. Dental cases show a considerable decrease, a fact that may be accounted for by the experience of patients proving that even at a hospital dentistry is not altogether painlessly performed. The report closes with a grateful acknowledgment of the Matron, Miss Tillett's, excellent services and economical management.

